# Soil bacterial community structure and functioning in a long-term conservation agriculture experiment under semi-arid rainfed production system

**DOI:** 10.3389/fmicb.2023.1102682

**Published:** 2023-06-15

**Authors:** G. Pratibha, M. Manjunath, B. M. K. Raju, I. Srinivas, K. V. Rao, Arun K. Shanker, J. V. N. S. Prasad, M. Srinivasa Rao, Sumanta Kundu, A. K. Indoria, Upendra Kumar, K. Srinivasa Rao, Shivakumar Anna, Ch. Srinivasa Rao, V. K. Singh, A. K. Biswas, S. K. Chaudhari

**Affiliations:** ^1^ICAR-Central Research Institute for Dryland Agriculture, Hyderabad, Telangana, India; ^2^ICAR-National Rice Research Institute, Cuttack, Odisha, India; ^3^ICAR-National Academy of Agricultural Research Management, Hyderabad, Telangana, India; ^4^ICAR-Indian Institute of Soil Science, Bhopal, Madhya Pradesh, India; ^5^Indian Council of Agricultural Research, New Delhi, India

**Keywords:** tillage, residues, soil microbial community, enzyme activities, soil organic carbon, nutrient availability

## Abstract

Soil microbial communities are important drivers of biogeochemical cycling of nutrients, organic matter decomposition, soil organic carbon, and Greenhouse gas emissions (GHGs: CO_2_, N_2_O, and CH_4_) and are influenced by crop and soil management practices. The knowledge on the impact of conservation agriculture (CA) on soil bacterial diversity, nutrient availability, and GHG emissions in semi-arid regions under rainfed conditions is vital to develop sustainable agricultural practices, but such information has not been systemically documented. Hence, studies were conducted for 10 years in rainfed pigeonpea (*Cajanus cajan* L.)—castor bean (*Ricinus communis* L.) cropping system under semi-arid conditions to assess the effects of tillage and crop residue levels on the soil bacterial diversity, enzyme activity (Dehydrogenase, urease, acid phosphatase, and alkaline phosphatase), GHG emissions, and soil available nutrients (Nitrogen, phosphorus, and potassium). Sequencing of soil DNA through Illumina HiSeq-based 16S rRNA amplicon sequencing technology has revealed that bacterial community responded to both tillage and residue levels. The relative abundance of Actinobacteria in terms of Operational Taxonomic Unit (OTUs) at phyla, class as well as genera level was higher in CA (NTR1: No Tillage + 10 cm anchored residue and NTR2 NT + 30 cm anchored residue) over CT (conventional tillage without crop residues). CA resulted in higher enzyme activities (dehydrogenase, urease, acid phosphatase, and alkaline phosphatase) and reduction in GHG emissions over CT. CA recorded 34% higher and 3% lower OC, as compared to CT, and CTR1, respectively. CA recorded 10, 34, and 26% higher available nitrogen, phosphorus, and potassium over CT and CTR1, respectively. NTR1 recorded 25 and 38% lower N_2_O emissions as compared to CTR1 and CTR2, respectively. Whereas only NT recorded 12% higher N_2_O emissions as compared to CT. Overall, the results of the study indicate that CA improves the relative abundance of soil bacterial communities, nutrient availability, and enzyme activities, and may help to contribute to the mitigation of climate change, and sustainability in rainfed areas.

## 1. Introduction

Two major serious concerns to meet the food demand of increasing population in developing countries are climate change and soil degradation. The resource intensive technologies like intense tillage, high yielding varieties, chemical fertilizers, and pesticides employed in the green revolution were productive and profitable but in long run, these practices were considered ecologically intrusive and environmentally unsustainable (Sial et al., 2021). Under the growing challenges of climate change, soil health deterioration may be exacerbated in the future, and the sustainability of natural resources may become a major concern. Intensive tillage is an important agronomic management practice for improving productivity in the short term since it improves the seedling establishment and reduces weed growth, but nearly 40% of agricultural lands have been degraded due to intensive tillage globally (Krauss et al., 2020). The reduction in soil carbon due to intensive tillage was also observed, this reduction is higher in the tropics because of higher temperature. The loss of soil biodiversity, increased soil compaction, runoff, soil erosion, and biotic pressure (pests, pathogens, and weeds) reflect the current degraded state of global soil health (Montgomery, 2007). Maintaining soil health is crucial to ensure food security to increasing human population in the long run, particularly in rainfed regions, where the soils are inherently poor in fertility and are subjected to frequent drought and erosion. Therefore, major transformation of the current agriculture practices is essential to maintain soil health and increase crop productivity.

To reduce the adverse impact of climate change and soil degradation there is an urgent need to develop and adopt climate-resilient rainfed technologies such as Conservation Agriculture (CA). The CA practices such as no tillage, crop rotation, and soil cover are considered efficient and environmentally friendly practices as they improve soil physical, chemical, and biological processes (Choudhary et al., 2018), reduce soil erosion, and improve soil quality which, in turn, help in increasing crop productivity. Besides, they also help in mitigating greenhouse gas emissions (GHGs) (Xiao et al., 2020). Among the GHG emissions, N_2_O is important gas since it contributes to 66% of the emissions from the agriculture sector. Both reduced tillage/zero tillage and crop residues incorporation recorded higher GHG mitigation potential apart from enhancing the energy use efficiency in rainfed pigeonpea-castor bean systems (Pratibha et al., 2015).

Soil health is key for sustainable agricultural production. SOC, nutrient availability were interlinked with soil microbiome. Microbial communities are important inhabitants of soil and play a major role in soil health as they mediate critical processes including biogeochemical cycling of carbon, nitrogen, and phosphorus which, in turn, improve soil fertility, and serve as an important reservoir for plant nutrients and improve the productivity of the terrestrial agroecosystems (Manjunath et al., 2015; Srinivasa Rao and Manjunath, 2017; Lehmann et al., 2020). Hence, maintaining the abundance and diversity of soil microorganisms is critical to sustain soil fertility (Malik et al., 2016; Trivedi et al., 2017). Microbial abundance and activity are correlated to carbon and nutrients of plant residues, they act as good early indicators of soil quality and productivity by quickly displaying the effects of soil and crop management practices in relation to physical and chemical properties of soil. In addition, the microbial population determines the sustainable productivity of agricultural lands, ecosystem resilience against nutrient mining, soil and water resources degradation, and GHG emissions (Vineela et al., 2008; Wagg et al., 2014). Hence, the knowledge of the impact of soil and crop management practices on microbial community structure and diversity is essential for assessing the effectiveness of management practices (Liang et al., 2016). Several reports are available on the influence of tillage, residue management, and cropping sequences on crop productivity, input use efficiency, soil carbon pools, soil physical properties (Alam et al., 2017), greenhouse gas (GHG) mitigation (Sapkota et al., 2019), and adaptation to climate risks (Jat et al., 2016) in irrigated conditions. However, such information on the effect of conservation agricultural practices on the composition, functioning of soil microorganisms, their structure, and role in the mitigation of GHG emissions and nutrient availability under semi-arid tropical rainfed ecosystems is very limited, particularly from long-term experiments in rainfed agriculture. Such studies in rainfed agriculture are important because globally 80% of the cultivated area is rainfed and it contributes to 60% of the world crop production. In India, it accounts for around 51% of the net sown area and 40% of total food grain production. These regions represent relatively low fertile, erosion-prone soils along with low SOC, high evapotranspiration (ET), and high soil temperature. Hence, crop productivity is also low under rainfed agriculture (Singh et al., 2020). Furthermore, globally the area under rainfed agriculture may further increase due to rising surface temperature as well as shifts in rainfall patterns (Maestre et al., 2015).

A field trial was conducted for 10 years and was initiated in 2009 at Hyderabad, which falls under the semi-arid rainfed regions, was used to study the influence of different tillage levels as well as crop residue levels on soil microbial diversity and community structure after continuous treatment for 10 years. The hypothesis of the present study was in rainfed semi-arid tropics CA practices, such as no tillage with residue retention (NT) influence soil microbial composition and diversity after 10 years. The main objectives of the study were (1) To assess the impact of different tillage practices and residue levels on soil bacterial communities, enzyme activities, nutrient availability and GHG mitigation potential in semi-arid rainfed production system.

## 2. Materials and methods

### 2.1. Experimental site

A long-term experiment was initiated at the Hayathnagar Research Farm (HRF) of the ICAR-Central Research Institute for Dryland Agriculture (ICAR-CRIDA), Hyderabad, India (17°23′ N latitude,78°29′ E longitude, 540 m above mean sea level). The experimental area falls under semi-arid climate with a mean annual rainfall of 750 mm, maximum and minimum temperature during the experimental period was 32°C and 20°C, respectively. Initial soil properties such as soil texture (Bouyoucos, 1962), SOC (Walkley and Black, 1934), nitrogen (Subbiah and Asija, 1956) and potassium (Van Reeuwijk, 2002) were estimated by collecting soil samples before the start of the experiment. The soil type of the experimental site was Typic Haplustalf with 72.8% sand, 8.2% silt, and 19% clay. The available N (KMnO_4_ extractable N contents), P, K, and SOC content of soil before beginning of the experiment were 156.8 kg ha^−1^, 15.59 kg ha^−1^, 179.2 kg ha^−1^, and 3.1 g kg^−1^, respectively.

### 2.2. Experimental details and climate

The long-term study was initially started in pigeonpea (*Cajanus cajan* L.)—castor bean (*Ricinus communis* L.) crop as an annual rotation during the rainy season starting from June to December. In the year 2009, pigeonpea was sown. The experiment was laid out in a split-plot design with three tillage systems as main plots (300 m^2^) and residue levels in subplots (100 m^2^) with three replications. Tillage treatments included conventional tillage (CT) of the region, it consisted of 3 passes of tillage, first pass was with a disc plow (15–20 cm depth) after summer showers during April/May, followed by two passes with cultivator and disc harrow after the onset of monsoon between second fortnight of June or first fortnight of July depending on the onset of monsoon just before sowing of the crop. Reduced tillage (RT) consisted of one pass of cultivator followed by disc harrowing before sowing with the onset of monsoon. No tillage was direct sowing without tillage (NT) and inter-cultivation operations were not done.

Tillage treatments in the main plots were split into three subplots. Initially the subplots comprised three harvest heights of pigeonpea and castor (anchored residues 0 cm, 10 cm, and 30 cm harvest height) till 2013. In the year 2014, dhaincha (*Sesbania rostrata*) was introduced as live mulch in residual plots (10 and 30 cm anchored residues) to increase the residues levels in the soil (Supplementary Figure S1). Five-meter buffer strips were maintained between the main plots. The crop was sown between the third week of June and the second week of July depending on the onset of the monsoon each year. Tractor drawn CRIDA precision planter was used to sow the crop (Pratibha et al., 2015). In this study, pigeonpea was sown in 2009, 2011, 2013, 2015, 2017, and 2019 and castor bean during the years 2010, 2012, 2014, 2016, and 2018. Pigeonpea and castor received a fertilizer (kg ha^−1^) of 20 N-60 P-60 K and 50 N-60 P-40 K, respectively. Cultivation practices adopted in different treatments were given in Pratibha et al. (2015) and briefly described in Supplementary Table S1.

### 2.3. Soil sampling and analyses

Soil samples (500 g) were collected from each treatment in three replications (0–15 cm depth) at the harvest of the castor bean crop in January 2020 (after completion of 5 pigeonpea-castor cycle, 11 years after experimental treatments were established). Four random samples were taken in each plot with a core sampler of 5 cm diameter, these samples were mixed thoroughly to prepare composite samples and passed through 2 mm sieves to remove litter. Each composite soil sample was divided into three parts one part of soil was air-dried and used for the chemical analysis. The second part of the sample was stored at 4°C for the enzyme analysis. The third part was stored at −20°C for DNA isolation and sequencing.

SOC and available nitrogen were estimated using a modified Walkley–Black wet oxidation method and Kjeldahl method, respectively. Available P was estimated by spectrophotometry based on the methodology developed by Olsen and Sommers (1982). Soil particle size distribution was determined using the Bouyoucos hydrometer method. Potassium was estimated by extracting with a 1 N solution of ammonium acetate at pH 7, the K^+^ estimation was done by flame emission spectrometry (Van Reeuwijk, 2002). Enzyme activities like dehydrogenase activity (Casida et al., 1964), urease activity (Tabatabai and Bremner, 1972), acid and alkaline phosphatase activities were estimated by following modified protocol as suggested by Deng et al. (2013), microbial biomass carbon (MBC) and microbial biomass Nitrogen (MBN) were determined by fumigation extraction method and calculated using conversion factors of 0.45 for MBC and 0.54 for MBN (Vance et al., 1987) measured in triplicate and expressed on a dry weight basis” retains the intended meaning and amend if necessary.

### 2.4. GHG flux measurements

GHG (CO_2_, N_2_O, and CH_4_) emissions measurements were done using the insulated static vented rectangular aluminum chambers (80 cm × 40 cm × 10 cm) of a cross-sectional area of 0.32 m^2^ with a height of 10 cm (Livingston and Hutchinson, 1995). The vented chamber has an anchor and a cover as two-piece system. The chambers were placed in a water channel that was welded on to anchors that were inserted 10 cm into the soil. Anchors were installed perpendicular to the crop row so that each chamber has the root system inside it. Installed anchors were retained in the field and were removed for tillage and planting operations and reinstalled near the initial locations. Gas samples were collected after 24 h of anchor installation to stabilize the anchor in the soil. GHG (CO_2_ and N_2_O flux) were measured at 3-d intervals during the first 4 months after planting. After 4 months gas sampling was done at 7 days interval till harvest as the crop growth rate was slow and precipitation events declined, further the effect of N fertilizer on N_2_O flux diminished due to N uptake by crop. The gas samples were collected between 9:00 and 11:00 a.m. with syringe at 0, 15, and 30 min after closing the top cover. The gas samples (60 mL to ensure over pressure of sample in the tubes) were injected into 20-ml vacuumized vials and gas samples were analyzed at CRIDA, Hyderabad, with a fully automated gas chromatograph (Model 4,200; Bruker Palo Alto, CA). This instrument was equipped with thermal conductivity, flame ionization, and electron capture detectors to analyze CO_2_, CH_4_, and N_2_O, respectively. Cumulative seasonal GHG fluxes were calculated from the linear or nonlinear increase in concentration (selected according to the emission pattern) in the chamber headspace with time (Livingston and Hutchinson, 1995).

### 2.5. Extraction and sequencing of soil DNA

The total genomic DNA was extracted from 250 mg of soil from each composite sample using the DNeasy Power soil Kit (Qiagen Pvt. Ltd., United States). Qubit Fluorometer (V.3.0) was used to estimate the DNA concentration. Specific V3 forward primer (CCTACGGGNBGCASCAG) and V4 reverse primer (GACTACNVGGGTATCTAATCC) were used to amplify the V3-V4 region of 16S rRNA amplified product was checked on 2% agarose gel and gel purification was done to remove nonspecific amplifications. Five nanograms of the amplified product were used for library preparation using the NEBNext Ultra DNA library preparation kit according to the manufacturer’s instructions. The library quantification and quality estimations were done using Agilent 2200 Tape Station. The prepared libraries were sequenced in Illumina HiSeq 2500 platform for 2 × 250bp read length.

The raw reads obtained from the Illumina sequencing platform after demultiplexing were subjected to the Fast QC program (Version.0.11.8) to check the quality of the reads with default parameters. Base quality (PhredScore; Q), base composition, GC content, ambiguous bases (other than A, T, G, C), and adapter dimers were thoroughly checked before the Bioinformatics analysis. Base quality of each cycle for all samples was recorded. More than 80% of the total reads have a phred score greater than 30 (>Q30; error-probability ≥0.001). The base composition of the left and right end of the paired-end read sequences is calculated. Since the target sequence is that of the V3-V4 region, sequence composition bias is observed in the sample. The average GC content distribution of the sequenced read of the samples was in the range of 30–60%. The forward V3 specific primer and reverse V4 specific primers were trimmed using an In-house PERL script. Properly paired-end reads with Phred score quality (*Q* > 20) were considered for V3-V4 consensus generation. Primer trimmed, high-quality paired-end reads were pair-wisely allowed to merge/stitch to get the V3-V4 amplicon consensus FASTA sequences. The reads were merged using the FLASH program (Version 1.2.11) with a minimum overlap of 10 bp to a maximum overlap of 240 bp with Zero mismatches. While making consensus V3-V4 sequence all consensus reads formed with an average contig length of 350 to 450 bp. The *de novo* chimera removal method UCHIME (version 11) implemented in the tool VSEARCH was used for removing Chimeras. The Operational Taxonomic Units (OTU) picking and taxonomy classification were assessed using the pre-processed consensus V3-V4 sequences (D’Argenio et al., 2014). Uclust program (similarity cuto. = 0.97) available in QIIME software was used to pool and cluster into OTUs based on their sequence similarity from pre-processed reads from all samples. A total of 273,250 OTUs were identified from 1,927,490 reads (1, 2). From 273,250 total OTUs, 247,819 OTUs with less than 5 reads were removed and 25,431 OTUs were selected for further analysis.

Quantitative insights into microbial ecology (QIIME1) program (Version: 1.9.1) was used for the entire downstream analysis (Caporaso et al., 2010). The representative sequences from each clustered OTUs were picked and aligned against the SILVA core set of sequences using the PyNAST program. Further, taxonomy classification was performed using the RDP classifier by mapping each representative sequence against the SILVA OTUs database. Total sequence reads ranged from 357125 to 569539 after filtering using QIIME (Quantitative Insights Into Microbial Ecology) quality filters with default settings (Supplementary Table S2).

The microbial diversity within the samples (Alpha diversity and rarefaction curves) was analyzed by calculating Shannon, Chao1, and observed species metrics. The chao1 metric assesses the species richness whereas the Shannon metric measures OTU abundances, and explains both richness and evenness. The observed species metric is the count of unique OTUs found in the sample. For beta diversity analysis of samples, the distance matrix was generated using both the weighted and unweighted UniFrac approach. Microbial diversity was compared using sequence abundances by taking Weighted UniFrac into account. For all the samples, a Jackknife test was done to construct a consensus UPGMA (Unweighted Pair Group Method with Arithmetic Mean) tree. The resulting consensus was taken for UPGMA trees built using weighted and unweighted UniFrac distance matrix.

### 2.6. Raw sequence data submission

The raw sequence data were deposited in the National Centre for Biotechnology Information (NCBI), New York as Sequence Reads Archive (SRA) with the Bio Project accession number PRJNA719998.

### 2.7. Plant growth-promoting bacteria

The genera such as *Azotobacter, Azospirillum, Bacillus, Pseudomonas, Alcaligenes, Arthrobacter, Burkholderia, Paenibacillus, Serratia, Klebsiella*, and *Achromobacter* were considered as their number was determined based on the percentage of total OTUs observed in the study.

### 2.8. Statistical analysis

The impact of tillage and crop residue levels on the soil nutrient and enzyme parameters were analyzed using SAS 9.2 version. The principal coordinate analysis (PCoA) of the data was done using facto extra package in R. Non-Metric multidimensional scaling (NMDS) based on Bray-Curtis distances was analyzed in R 3.6.1 with packages vegan 2.5–5 and phylo seq to study the distribution patterns of N cycling functional groups and their activity in tillage and residue levels. R with the function anosim in package vegan was used for the analysis of similarities (ANOSIM), a rank-based nonparametric statistical test to compare groups and test the null hypothesis whether the similarity between groups is higher than or equal to the similarity within the groups. The function Tukey HSD betadisper in package vegan was used to calculate Tukey’s honest significant differences between groups. In this investigation, a *p* value of 0.05 was accepted for the statistically significant difference unless otherwise noted.

The principal component analysis (PCA) was done with 24 attributes following Andrews et al. (2002) and Choudhary et al. (2018). The principal components (PC) having high eigenvalues and variables with high factor loading were considered to be variables that best represented system attributes. Hence, only the PCs with eigenvalues >0.75 and which explained at least 5% of the variation in the data were examined. Only highly weighted variables within each PC were considered for the minimum data set (MDS). In a single PC, if more than one variable was retained, multivariate Pearson’s correlation coefficients were used to determine if the variables could be considered redundant. Variables with the highest correlation sum were selected for the MDS.

## 3. Results

### 3.1. Soil bacterial community structure and diversity analysis

**Figure 1:**
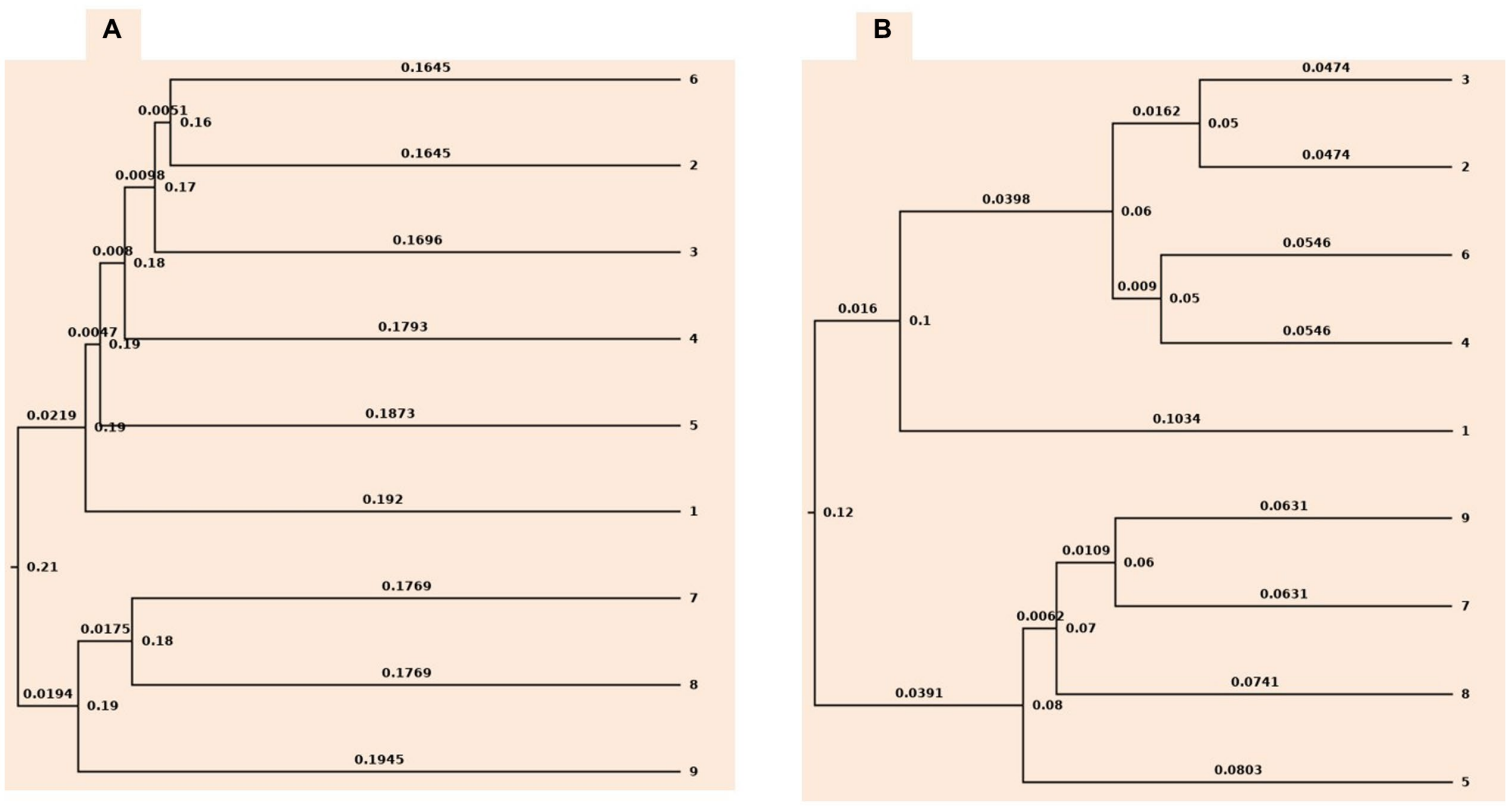
Cluster tree based on **(A)** unweighted unifrac approach **(B)** weighted unifrac approach. 1-conventional tillage; 2-conventional tillage +10 cm anchored residue; 3-conventional tillage +30 cm anchored residue; 4-reduced tillage; 5-reduced tillage +10 cm anchored residue; 6-reduced tillage +30 cm anchored residue; 7-zero tillage; 8-zero tillage +10 cm anchored residue; and 9-zero tillage +30 cm anchored residue.

Rarefaction and ChaoI analysis were performed using bacterial operational taxonomic units (OTUs) vs. sequences obtained in each treatment (Supplementary Figure S2). In addition, UPGMA clustering analysis based on weighted and unweighted unifrac distance showed that the bacterial communities were influenced by different levels of tillage *viz.*, CT, RT, and NT, and also residue levels based on their cluster pattern (Figure 1).

### 3.2. Influence of tillage and crop residue incorporation on the composition of soil bacterial communities

#### 3.2.1. Relative proportion of bacterial OTUs at phyla levels

**Figure 2:**
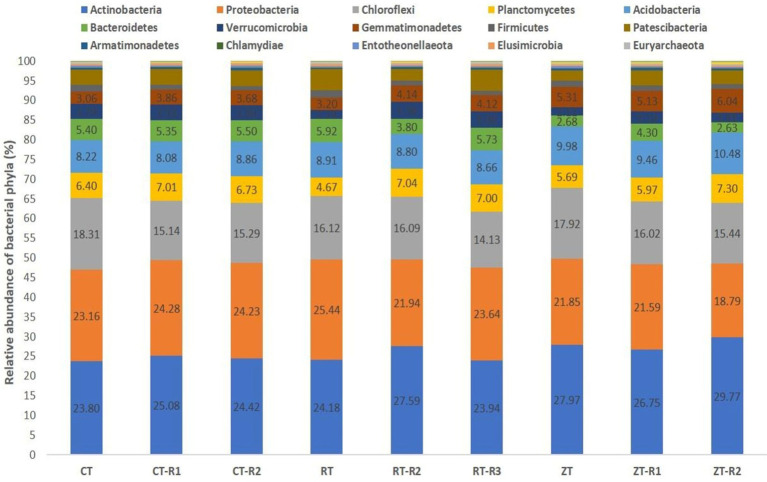
Effect of tillage and crop residues on the composition of bacterial phyla under semi-arid rainfed production system. CT-conventional tillage; CTR1-conventional tillage +10 cm anchored residue; CTR2-conventional tillage +30 cm anchored residue; RT-reduced tillage; RTR1-reduced tillage+10 cm anchored residue; RTR2-reduced tillage 30 cm anchored residue; NT-zero tillage; NTR1-zero tillage +10 cm anchored residue; and NTR2-zero tillage +30 cm anchored residue.

A total of 34 phyla were observed in different tillage and residue management. In current study, the dominant bacterial phyla were Actinobacteria, Proteobacteria, Chloroflexi, Planctomycetes, Acidobacteria, Bacteroidetes, Verrucomicrobia, and Gemmatimonadetes, accounting for around 85% of reads represented by the operational taxonomic units (OTUs) (Figure 2). In addition, Euryarchaeota, Nitrospirae, Thaumarchaeota, Thermotogae, Deinococcus-Thermus, Omnitrophicaeota, Fibrobacteres, Dependentiae, Rokubacteria, and Latescibacteria were detected in all the samples with low abundance of OTUs. In our study, the Actinobacteria were the dominant bacteria and the relative proportion of Actinobacteria ranged from 23.86 to 29.30% across all treatments.

**Figure 3:**
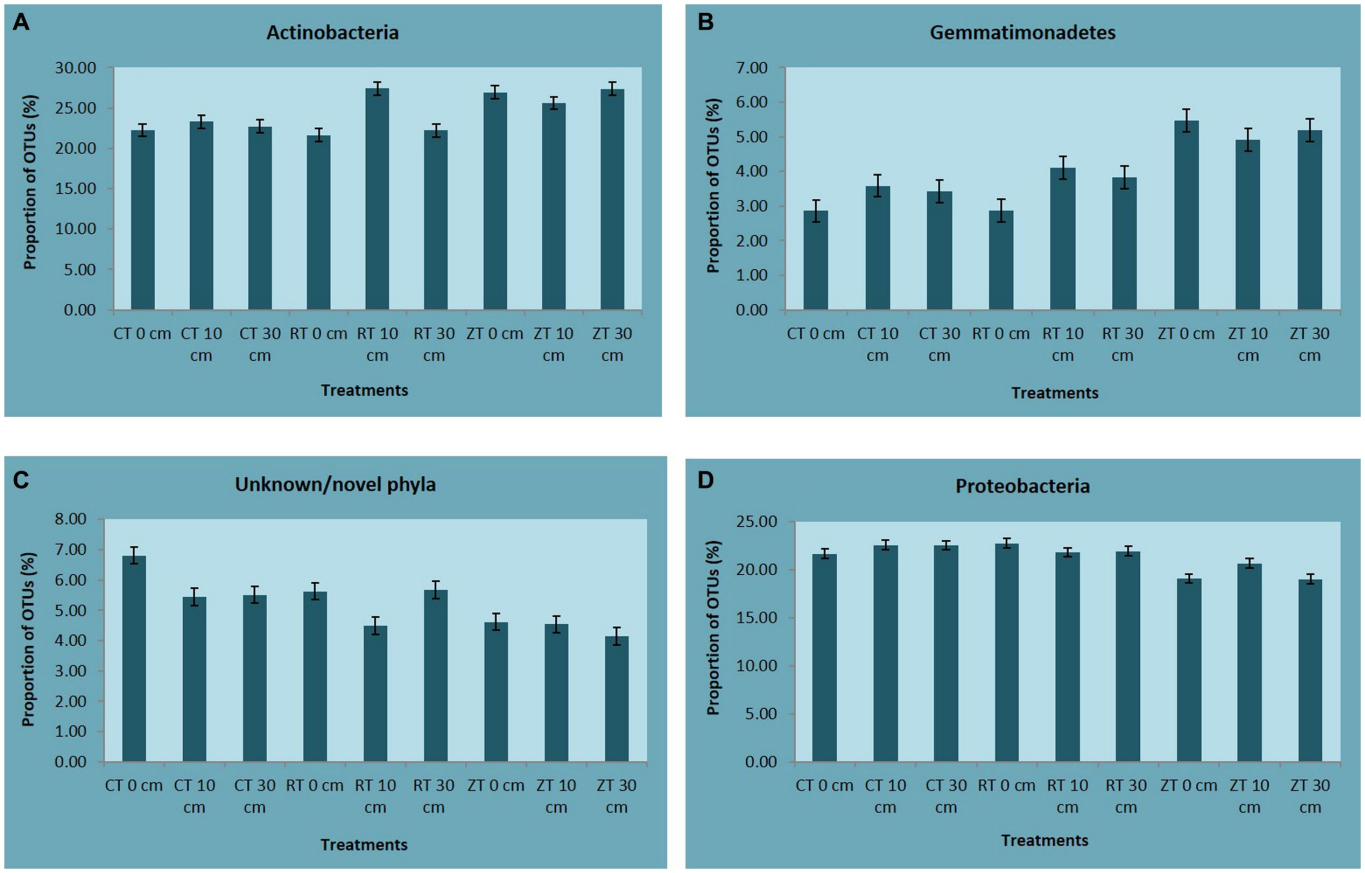
The relative abundance of **(A)** Actinobacteria, **(B)** Gemmatimonadetes, **(C)** Unknown/novel phyla, and **(D)** Proteobacteria. CT-conventional tillage; CTR1-conventional tillage +10 cm anchored residue; CTR2-conventional tillage +30 cm anchored residue; RT-reduced tillage; RTR1-reduced tillage+10 cm anchored residue; RTR2-reduced tillage +30 cm anchored residue; NT-zero tillage; NTR1-zero tillage +10 cm anchored residue; and NTR2-zero tillage +30 cm anchored residue.

In the present investigation, the intensity of tillage and residue levels influenced the abundance of the different bacterial communities. Among the tillage treatments averaged over the residue levels, NT recorded higher total abundance and 21, 23, 50, and 33% higher relative abundance of bacterial phyla like Actinobacteria, Acidobacteria, Gemmatimonadetes, and Nitrospirae, respectively, over CT. While CT and RT recorded a higher abundance of copiotrophic bacteria such as Proteobacteria, Chloroflexi, Planctomycetes, Bacteroidetes, Verrucomicrobia, and Firmicutes. The relative proportion of Actinobacteria ranged from 23.80 to 29.76% across all treatments. This was maximum in NTR2 (29.76%) and was minimum in CT (23.8%) (Figure 3A). NT (5.49%) recorded a higher relative abundance of Gemmatimonadetes as compared to CT (3.53%) and RT (3.81%) (Figure 3B). Oligotrophic bacteria, like Acidobacteria, and Nitrospira, were more predominant in NT. Novel/unknown/unclassified phyla accounted for about 4–7% of total OTUs in the respective samples, they were maximum in CT followed by RT and NT (Figure 3C). The relative abundance of Proteobacteria ranged from 20.22 to 26.67% (Figure 3D). In present study, the maximum population of Proteobacteria and the members of different Proteobacteria classes such as alpha (α), gamma (γ), and delta (δ) were observed in CT averaged across residue treatments. However, no significant differences observed between CT and RT (Supplementary Figure S3). The higher bacterial abundance with pigeonpea and castor bean residues along with dhaincha live mulch residues averaged over tillage practices was observed over no residues. The higher total and relative abundance of Actinobacteria was observed in higher residue levels as compared to no residues. The dominant bacterial groups differed between tillage and residue levels. NTR2 and RTR1 recorded a higher abundance of Actinobacteria as compared to CT. Nitrospirae, the bacterial phylum containing ammonia-oxidizing and nitrate-oxidizing bacteria, was significantly higher in NTR as compared to CTR and RTR.

#### 3.2.2. Relative proportion of bacterial OTUs at class and genus levels

In the present study a total of 94 classes of bacteria were observed. The relative abundance of the Actinobacteria class was highest in RTR1, followed by Acidimicrobiia and Thermoleophilia in NTR1 and NTR2 (Supplementary Figure S4). In our study, functionally diverse bacterial genera such as Geodermatophilus, Bacillus, Streptomyces, and methylotrophic Methylobacterium were observed. Among the genera of Actinobacteria, Streptomyces, Nocardioides, and Pseudonocardia were predominant (Supplementary Figure S5).

#### 3.2.3. Plant growth-promoting bacteria (PGPR)

Among the plant growth-promoting bacteria (PGPR) observed in this study, *Bacillus*, *Paenibacillus* and *Pseudomonas* were predominant. The total abundance of PGPR was significantly influenced by tillage and residue levels. NT recorded the highest PGPR as compared to CT and RT. The residue levels positively influenced the abundance of PGPR. NTR1 and NTR2 recorded 28 and 5% higher relative abundance of *Bacillus* over CT and NT. *Klebsiella* was more abundant in NTR1 and NTR2 as compared to CT, RT, CTR1, CTR2, and RT. The maximum population of *Pseudomonas* was observed in RTR1 and NTR1. The Arthrobacter population was more in RTR1. Relative abundance of *Azotobacter* was highest in CT (Supplementary Figure S6).

The major ammonia oxidizing bacteria (AOB) considered in the study were *Nitrosomonas*, *Nitrosospira* and *Nitrosococcus*. Among them, *Nitrosospira* was predominant and constituted around 70% of AOB. The AOB was significantly influenced by the intensity of tillage and residue application. Total abundance of AOB in NT was 32 and 29% higher than in CT and RT, respectively. Whereas among the different AOB, *Nitrosomonas*, the β -Proteobacteria phylum was 72 and 65% higher in CT as compared to NT and RT, respectively. Whereas, other bacteria like *Nitrospira* and *Nitrolancea* (nitrite-oxidizing bacteria) were higher in NT as compared to CT (Supplementary Figure S7). NTR1 and NTR2 recorded significantly higher ammonia-oxidizing and nitrite-oxidizing bacteria like, *Nitrospira*, and *Nitrolancea* as compared to CT. Residue addition recorded higher *Nitrosomonas* and the same was not observed in no residue applied treatments at all tillage levels.

### 3.3. Biplot analysis

**Figure 4:**
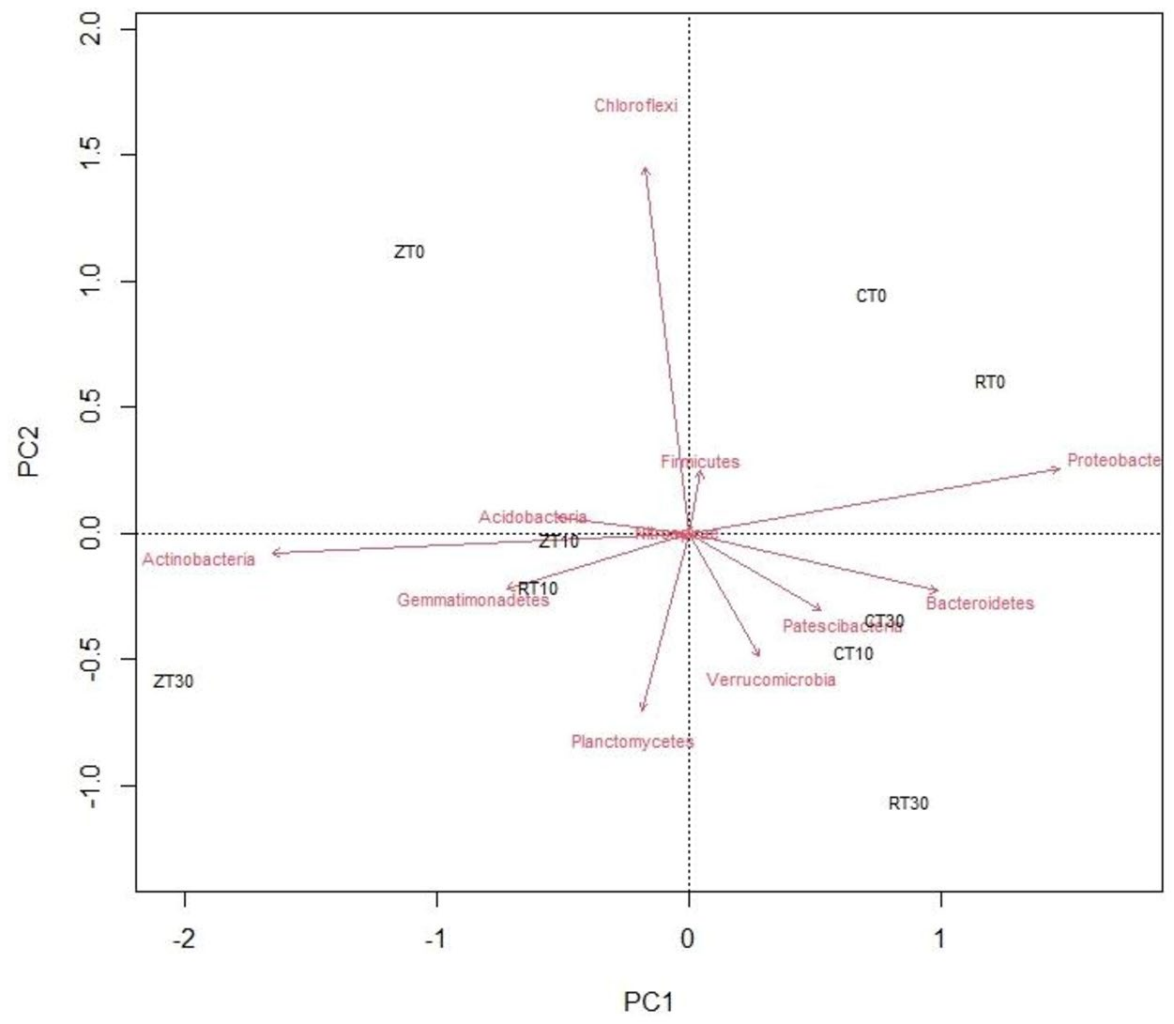
Biplot analysis depicting the association between different conservation agricultural practices and bacterial phyla. CT-conventional tillage; CTR1-conventional tillage +10 cm anchored residue; CTR2-conventional tillage +30 cm anchored residue; RT-reduced tillage; RTR1-reduced tillage+10 cm anchored residue; RTR2-reduced tillage +30 cm anchored residue; NT-zero tillage; NTR1-zero tillage +10 cm anchored residue; and NTR2-zero tillage +30 cm anchored residue.

A PCoA was done by calculating the Bray–Curtis dissimilarity index among the samples for improved overview of the microbial communities and their relationships with different tillage and residue addition (Figure 4). Principal component analysis could clearly reflect the variations between different tillage and residue treatments with regard to soil bacterial communities. First and second axes explained 56.20 and 24.70% of the total variation, respectively. CT was well separated from other treatments such as RTR1, NTR1, and NTR2. Majority of the bacteria *viz.*, Verrucomicrobia, Patescibacteria, and Bacteroidetes, were distributed in treatments with residue incorporation, i.e., CTR2, CTR1, and RTR2. The Actinobacteria, Gemmatimonadetes, Planctomycetes, and Acidobacteria were distributed in zero tillage treatments (Figure 4).

### 3.4. Soil nutrients and enzyme activities

In the present study, after 10 years SOC increased with decrease in the intensity of tillage practices. Zero tillage (NT) recorded higher SOC (11.14 g/kg) as compared to reduced tillage (RT) [9.66 g/kg] and conventional tillage (CT) [8.4 g/kg] averaged over residues. Addition of crop residues through manipulation of harvest height of the crops up to 10 cm (R1) and 30 cm (R2) along with dhaincha live mulch increased the SOC by 26 and 24% as compared to no residues, respectively. NTR1 (NT + 10 cm anchored residues) [11.72 g/kg] and NTR2 (12.5 g/kg) [NT + 30 cm anchored residue] recorded significantly higher SOC content as compared to NT (Zero tillage without residues) or CT with 10 and 30 cm residues (CTR 1, CT R2) and without residues (CT) (Table 1).

**Table 1:** Influence of different tillage and residue levels on soil available nutrients (kg ha^−1^), SOC and GHG emissions.

Tillage	Residue levels	Available nutrients (kg ha^−1^)			SOC (g kg^−1^)	GHG emissions (kg ha^−1^season^−1^)		
		Nitrogen	Phosphorus	Potassium		CO_2_	N_2_O	CH_4_
CT	0	154.62^A^	11.88^A^	144.03^A^	6.12^A^	2326.00^C^	0.37^AB^	0.20^E^
	R1	173.00^CD^	12.87^B^	165.24^B^	9.40^BC^	3391.00^E^	0.47^E^	0.16^CD^
	R2	181.92^DE^	13.94^CD^	170.00^BC^	9.70^C^	3450.00^E^	0.57^F^	0.11^B^
RT	0	158.89^AB^	12.99^B^	162.89^B^	8.58^B^	1976.00^A^	0.37^AB^	0.14^CD^
	R1	179.00^DE^	13.14^BC^	172.00^BC^	10.20^C^	2359.00^BC^	0.46^DE^	0.15^D^
	R2	185.00^E^	14.95^E^	183.89^CD^	10.20^C^	2413.00^BC^	0.43^CDE^	0.15^D^
NT	0	167.00^BC^	13.83^BC^	157.17^B^	9.20^BC^	2071.00^AB^	0.42^CD^	0.12^BC^
	R1	199.00^F^	16.53^F^	174.23^C^	11.70^D^	2294.00^BC^	0.35^A^	0.03^A^
	R2	200.00^F^	18.22^G^	194.47^D^	12.50^E^	2637.00^D^	0.40^BC^	0.03^A^
Mean	CT	169.85	12.89	159.76	8.40	3056.00	0.47	0.15
	RT	174.30	13.69	172.92	9.66	2250.00	0.42	0.14
	NT	188.67	16.19	175.29	11.14	2334.00	0.39	0.06
Residue levels	0	160.17	12.90	154.70	7.96	2124.00	0.38	0.15
	R1	183.67	14.18	170.49	10.44	2681.00	0.42	0.11
	R2	188.98	15.70	182.78	10.80	2834.00	0.47	0.09

Significant interaction between tillage and residue levels, was observed means followed by the same letter are not significantly different at *p* = 0.05; CT, conventional tillage; RT, reduced tillage; NT, zero tillage; No residue:0 cm, 1:10 cm harvesting height + dhaincha live mulch and 2: 30 cm harvesting height from ground + dhaincha live mulch.

The pH of the soil was not significantly influenced by tillage practices or residue levels. The soil available nitrogen, phosphorus, and potassium were significantly (*p* < 0.05) influenced by tillage and residue levels after 10 years. NT averaged over anchored residues recorded significantly higher available nitrogen (188.66 kg ha^−1^), phosphorus (16.19 kg ha^−1^), and potassium (175.29 kg ha^−1^) as compared to CT and RT, but CT and RT were at par with each other. Whereas the available phosphorus in NT was 20 and 15% higher over CT and RT, respectively (Table 1). The available macronutrients are influenced by residue application. 10 cm and 30 cm residue application led to significantly higher nutrients as compared to no residues but 10 and 30 cm were at par with each other.

The available macro nutrients nitrogen, phosphorus, and potassium were significantly higher in NTR1 (199 kg ha^−1^ N, 16.53 kg ha^−1^ P, 174.24 kg ha^−1^ K) and NTR2 (200 kg ha^−1^ N, 18.22 kg ha^−1^ P,194.47 kg ha^−1^ K) as compared to CT, NT, RT, CTR1, CTR2, and RTR1. Whereas the available nutrients were higher in RT and CT with different residue levels as compared to CT and RT.

The soil enzyme activities like phosphatase, dehydrogenase, and urease which are of microbial origin are involved in the soil biological processes and nutrient cycling hence these are considered as better indicators of soil health. In the present study, NT averaged over crop residues recorded higher acid phosphatase (5.44 μg p-nitrophenol/g soil/h), alkaline phosphatase (10.12 μg p-nitrophenol/g soil/h), and dehydrogenase activity (24 μg TPF/g soil/h) as compared to CT and RT averaged over residues, respectively. The R1 and R2 residues recorded significantly higher acid phosphatase, alkaline phosphatase, and higher dehydrogenase activity as compared to R0. NTR2 recorded significantly (*p* < 0.05) higher enzyme activities *viz.*, dehydrogenase (3.24 μg TPF/g soil/h), acid phosphatase (7.08 μg p-nitrophenol/g soil/h), alkaline phosphatase (16.15 μg p-nitrophenol/g soil/h) and urease (3.24 μg NH_4_/g soil/h) activities as compared to CTR1 and RTR1 (Table 2).

**Table 2:** Effect of tillage and residue levels on enzyme activities.

Tillage	Residue levels	Dehydrogenase	Urease	Acid phosphatase	Alkaline phosphatase	MBC	MBN
		(μg TPF g^−1^ soil h^−1^)	(μg NH_4_ g^−1^ soil h^−1^)	(μg p-nitrophenol g^−1^ soil h^−1^)		(μg g^−1^ soil)	
CT	0	1.35^A^	13.28^A^	2.93^AB^	6.06^A^	129.23^AB^	46.70^B^
	R1	1.58^B^	14.53^BC^	FIGURE 3.94^BCD^	8.23^BCD^	156.36^BC^	58.91^D^
	R2	1.56^B^	14.83^BC^	3.63^ABC^	7.31^BC^	161.53^C^	64.19^E^
RT	0	1.89^C^	13.91^AB^	2.57^A^	8.78^D^	129.55^AB^	46.81^B^
	R1	2.06^C^	14.06^AB^	5.09^DE^	8.77^D^	146.68^BC^	54.90^C^
	R2	2.05^C^	15.30^CD^	5.63^E^	6.92^AB^	142.17^B^	63.30^E^
NT	0	2.07^C^	14.15^AB^	4.53^C^	7.04^ABC^	125.70^A^	41.26^A^
	R1	2.59^D^	14.61^BC^	5.57^E^	10.15^E^	143.00^B^	49.47^B^
	R2	3.24^E^	16.20^D^	7.08^F^	16.15^F^	184.85^D^	59.31^D^
Mean	CT	1.49	14.21	3.50	7.20	149.00	56.60
	RT	2.20	14.42	4.43	8.15	138.00	55.04
	NT	2.63	14.98	5.73	11.13	154.00	50.01
Residue levels	0	1.77	13.78	3.34	7.29	128.00	44.92
	1	2.07	14.40	4.86	9.05	153.00	54.42
	2	2.48	15.44	5.44	10.12	161.00	62.28

Significant interaction between tillage and residue levels, was observed means followed by the same letter are not significantly different at *p* = 0.05; CT, conventional tillage; RT, reduced tillage; NT, zero tillage; No residue:0 cm, 1:10 cm harvesting height + dhaincha live mulch and 2: 30 cm harvesting height from ground + dhaincha live mulch.

### 3.5. Soil GHG emissions

Tillage and residue levels significantly influenced CO_2_ emissions. Cumulative CO_2_ emissions ranged from 1975 to 3,450 kg ha^−1^ season^−1^ were by CT (3,056 kg ha^−1^ season^−1^) averaged over crop residues recorded higher CO_2_ emissions as compared to RT and NT, respectively (Table 1). The CO_2_ emissions significantly increased with residue application and ranged between 2,124 to 2,884 kg ha^−1^ season^−1^. R2 and R3 recorded 20 and 25% higher emissions as compared to R1, respectively. CTR1 (3,391 kg ha^−1^ season^−1^) and CTR2 (3,450 kg ha^−1^ season^−1^) recorded significantly higher CO_2_ emissions.

Dryland soils normally act as sinks for atmospheric CH_4_. In the present study, seasonal cumulative CH_4_ emissions were positive but were very low (0.0026–0.195 kg ha^−1^ season^−1^) (Table 1). The methane flux was influenced by tillage and residue levels. It was negligible in zero tillage. NTR1 and NTR2 recorded the lowest methane fluxes.

The N_2_O emissions were significantly influenced by intensity of tillage and residue management. In this study, NT averaged over crop residues recorded 17% lower N_2_O emissions than CT. Crop residue application averaged over tillage practices significantly reduced the N_2_O emissions. R2 and R3 recorded 11 and 20% lower N_2_O emissions as compared to R0, respectively. The CTR1, CTR2, RTR1, RTR2 NTR2, and NTR1 recorded higher N_2_O emissions as compared to CT, RT, and NT, respectively (Table 1). NTR1 recorded 25 and 38% lower N_2_O emissions as compared to CTR1 and CTR2, respectively. Whereas NT recorded 12% higher N_2_O emissions as compared to CT.

### 3.6. Relation between bacterial phyla, soil available nutrients, and GHG fluxes

Changes in microbial population significantly influence nutrient cycling and other processes directly related to agricultural sustainability and greenhouse gas emissions such as nitrification, denitrification (N_2_O emissions), and CH_4_ oxidation.

Relation between soil bacterial phyla (top 11), soil available nutrients (N, P, and K), and SOC were estimated with principal component analysis (PCA). Four principal components (PCs) with eigenvalues >0.75 were extracted that together represented 91.81% of the total variance (Table 3). The PCA indicated that axis 1 (PC1), axis 2 (PC2), axis 3 (PC3), and axis 4 (PC4) showed 52.81, 21.62%, 10.1, 7.26 of the total variance, respectively (Supplementary Table S3). PC1 had the highest eigenvalue (14.244), and there were 15 variables (Actinobacteria, Acidobacteria, Gemmatimonadetes, Nitrospirae, acid phosphatase, alkaline phosphatase, dehydrogenase, urease, available P, N, K, SOC, and MBC) with positive loadings of eigen vector >0.75 and two negative loadings for bacterial communities, i.e., Chloroflexi and Firmicutes. PC2 had an eigenvalue of 7.136 and explained an additional 21.62% of the variability with positive loadings for Proteobacteria, Planctomycetes, Verrucomicrobia, N_2_O, and no negative loadings. The eigenvalue for PC3 was 3.805 with an additional 10.11% explanation of the total variability, showing a single positive loading for Patescibacteria and a single negative loading for CO_2_ emissions. The eigenvalue of PC4 was 2.45 explaining an additional 7.26% of the variability. PC4 showed single positive loadings for CH_4_.

**Table 3:** Relation between bacteria, available nutrients, SOC, and GHG emissions under different tillage and residue levels.

	AP	ALP	Dehydrogenase	Urease	MBC	MBN	OC	N	P	K	CO_2_	N_2_O	CH_4_
Actinobacteria	0.77^**^	0.58^**^	0.63^**^	0.73^**^	0.72^**^	0.55^**^	0.88^**^	0.79^**^	0.61^**^	0.83^**^	0.30	0.35	−0.65^**^
Proteobacteria	−0.52^**^	−0.57^**^	−0.47^*^	−0.17	−0.39^*^	0.41^*^	−0.29	−0.28	−0.49^**^	−0.12	0.38	0.40^*^	0.57^**^
Chloroflexi	−0.84^**^	−0.89^**^	−0.90^**^	−0.93^**^	−0.73^**^	−0.44^*^	−0.85^**^	−0.78^**^	−0.91^**^	−0.92^**^	−0.06	−0.05	0.84^**^
Planctomycetes	0.43^*^	0.07	0.15	0.52^**^	0.41^*^	0.89^**^	0.33	0.48	0.17	0.52^**^	0.68^**^	0.63^**^	−0.04
Acidobacteria	0.70^**^	0.56^**^	0.78^**^	0.86^**^	0.68^**^	−0.47^*^	0.88^**^	0.80^**^	0.81^**^	0.89^**^	−0.11	−0.220	−0.82^**^
Bacteroidetes	−0.54^**^	−0.53^**^	−0.46^*^	−0.19	−0.40^*^	0.40^*^	−0.37	−0.28	−0.45^*^	−0.16	0.32	0.25	0.63^**^
Verrucomicrobia	−0.03	−0.41^*^	−0.28	−0.03	0.07	0.65^**^	−0.08	0.09	−0.31	0.07	0.51^**^	0.52^**^	0.45^*^
Gemmatimonadetes	0.92^**^	0.67^**^	0.85^**^	0.79^**^	0.81^**^	0.22	0.89^**^	0.85^**^	0.88^**^	0.77^**^	0.03	−0.02	−0.95^**^
Firmicutes	−0.74^**^	−0.35	−0.48^*^	−0.73^**^	−0.73^**^	−0.65^**^	−0.7^**^	−0.76^**^	−0.58^**^	−0.68^**^	−0.49^*^	−0.61^**^	0.62^**^
Nitrospirae	0.92^**^	0.67^**^	0.89^**^	0.71^**^	0.76^**^	0.11	0.85^**^	0.85^**^	0.92^**^	0.71^**^	−0.17	−0.17	−0.94^**^

AP, acid phosphatase; ALP, alkaline phosphatase; P, available phosphorus; N, available nitrogen; K, available potassium; SOC, organic carbon; MBC, microbial biomass carbon; MBN, microbial biomass nitrogen. **significance at the *p* < 0.01, *significance at the *p* < 0.05.

To avoid redundancy, a correlation study (Pearson’s correlation) was done among the different variables. A positive correlation between SOC and SMBC indicated that SOC and SMBC are interlinked and they both together improved the microbial abundance, enzyme activities, and soil quality with management practices. A correlation study (Pearson’s correlation) among all the 24 variables was done and the findings revealed that the main bacterial phyla were correlated (positively or negatively) with soil available nutrients and enzymes (Table 3). In particular, SOC was significantly positively correlated with Actinobacteria (*r* = 0.88, *p* < 0.05), Acidobacteria (*r* = 0. 88, *p* < 0.05), Gemmatimonadetes (*r* = 0.89), and Nitrospirae (*r* = 0.85, *p* < 0.05) whereas negatively correlated with Chloroflexi (*r* = −0.85, *p* < 0.05) and Firmicutes (*r* = −0.70, *p* < 0.05). The other bacterial phyla did not show any significant relation. The available nutrients (NPK) were significantly positively correlated with Actinobacteria, Acidobacteria, Gemmatimonadetes and negatively correlated with Chloroflexi, and Firmicutes.

Enzyme activity was positively correlated with the bacterial population and available nutrients. Available nitrogen, phosphorus, potassium, and enzyme activities were strongly positively correlated with the phylum Actinobacteria, Gemmatimonadetes, and Nitrospira. Whereas the available N, P, and K and enzyme activities were significantly negatively correlated with Chloroflexi and Firmicutes. Acidobacteria was significantly correlated with available P and nitrogen (Chen et al., 2021). Acid phosphatase, alkaline phosphatase, dehydrogenase, and urease enzyme activities were significantly positively correlated with SOC, available nitrogen, phosphorus, and potassium.

A strong correlation was observed between N_2_O emissions and β- or γ-proteobacteria (AOB), Planctomycetes, and Firmicutes. Methane absorption was observed in this study and methane flux was negatively correlated with the abundance of Methylobacterium and was also positively correlated with Actinobacteria, Proteobacteria Chloroflexi, Acidobacteria, Bacteroidetes, Gemmatimonadetes and Firmicutes, and Nitrospirae.

## 4. Discussion

### 4.1. Influence of tillage and residues on soil bacterial community and structure

In the current study, we analyzed the effects of different tillage and crop residue management practices on soil bacterial communities. Since these practices influences soil environment including creation of favorable soil physico-chemical conditions like soil moisture, accumulation of soil organic matter (SOM), and available nutrients (Li et al., 2018), which, in turn, affects the relative abundance of the soil bacteria and their functions (Dong et al., 2017; Li et al., 2020a). In the present study, the dominant bacterial phyla across different treatments were Actinobacteria, Proteobacteria, Chloroflexi, Planctomycetes, Acidobacteria, Bacteroidetes, Verrucomicrobia, and Gemmatimonadetes (Figure 2). These bacteria thrive even under soil moisture deficit conditions (DeBruyn et al., 2011; Rehákov et al., 2015; Tyler, 2019). Whereas, long-term conservation agriculture experiment in Indo-Gangetic plains reported the predominance of Proteobacteria, Acidobacteria, Actinobacteria, and Bacteroidetes which represented >70% of the identified phyla (Choudhary et al., 2018, 2020). Proteobacteria are Gram-negative bacteria with many plant growth-promoting genera, use a wider range of C substances (Philippot et al., 2013). These bacteria play a major role in the biogeochemical cycle of plant nutrients and are also efficient decomposers of organic matter (Shanmugam et al., 2021). The higher abundance of bacterial communities such as Bacteroidetes and Proteobacteria, Planctomycetes and Gemmatimonadetes with residue application might be due to increase in nutrients supply. These bacterial communities are mainly involved in C or N cycling (Tang et al., 2019; Luan et al., 2020).

The lower soil disturbance and residues on the soil in NTR1 and NTR2 have provided a stable soil microenvironment for microbial growth, by moderating soil moisture, temperature, and soil organic matter enrichment (Choudhary et al., 2018; Akhtar et al., 2019). The higher total abundance and relative abundance of oligotrophic bacterial phyla like Actinobacteria, Acidobacteria, Gemmatimonadetes, and Nitrospira in NT as compared to CT and RT are because these bacteria have greater efficiency of scavenging nutrients from recalcitrant OM substrates (Wang et al., 2020). While higher abundance of copiotrophic bacteria like Proteobacteria, Chloroflexi, Planctomycetes, Bacteroidetes, Verrucomicrobia, and Firmicutes in CT is due to availability of easily decomposable organic material and available nutrients (Trivedi et al., 2017), the higher availability of organic matter due to inversion of organic matter to the top layers and close contact of organic material to microorganisms due to the breaking of aggregates with tillage (Dong et al., 2017; Sun et al., 2018; Zhu et al., 2018; Pan et al., 2020).

### 4.2. Linking soil bacteria with SOC and soil available nutrients

In rainfed semi-arid tropical climatic conditions, the SOC sequestration is challenging because of higher ambient temperature and low rainfall, since these two conditions favor faster chemical oxidation. Under these circumstances, CA (NT with crop residues) is an important viable option for SOC sequestration, mitigation of climate change, and improving crop productivity (Lal, 2015; Lal, 2016). The slow decomposition of organic carbon, redistribution of SOC within aggregates (Li et al., 2020b; Yang et al., 2022) due to lack of or minimum soil disturbance, higher soil aggregation and aggregate stability increased the SOC in NT averaged over crop residues by 29%. Whereas in CT, plowing caused the breakdown of soil aggregates, increased aeration, and thereby enhanced organic matter decomposition. This higher decomposition rate of crop residues or organic matter in CT is also supported by higher CO_2_ flux in CT (Table 1). Therefore, observations in this study confirm the notion that NT records higher soil C storage by reducing microbial CO_2_ respiration, through reduced oxidative stress and enhanced enzymatic transformation of organic material. The increased carbon input through residue addition does not increase only microbial diversity but also improve soil C accumulation and improve the soil nutrient cycling and soil enzyme activities and improve the nutrient availability. This, in turn, may stimulate soil C storage by promoting plant growth and soil C input (Lu et al., 2011; Huang et al., 2020) the increase in microbial diversity and MBC may stimulate the secretion of microbial by products and the formation of microbial necromass, which contributes to the stable soil C pool (Prommer et al., 2020). In present study, NT averaged over crop residues recorded 39 and 23% higher acid phosphatase, 35 and 27% alkaline phosphatase, 43 and 16% higher dehydrogenase as compared to CT and RT, respectively. This differential enzyme activity in different tillage practices was due to differences in the degree of soil disturbance (Zuber and Villamil, 2016; Zuber et al., 2018). Higher phosphatase and urease enzyme activity with the addition of crop residues is due to addition of easily degradable dhaincha live biomass which contains amino acids and carbohydrates and these provide more nutrients for microbial growth and activity (Malobane et al., 2020). This higher enzyme activity increases mineralization of nutrients which, in turn, increase the available nutrients this favors the microbial growth. Higher phosphatase activity in NTR1 led to enhancement in available phosphorus as these enzymes play a significant role in P bioavailability from native organic P compounds (Chavarría et al., 2016).

### 4.3. Soil GHG emissions

CA is an effective mitigation strategy and improves the soil functionality (Lal, 2004; Alvarez et al., 2014). However, reduced GHGs emissions in CA practice are still debatable. Some studies reported that CA significantly increased GHGs emissions (Oorts et al., 2007), whereas some studies revealed that, GHGs emissions were significantly reduced under NT and NTR as compared to CT (Rutkowska et al., 2018). Long-term studies on CA significantly change the GHGs concentration through SOM stocks, soil physico-chemical properties, and microbial composition as well as population.

The better substrate availability to microbes due to residue application, increased soil aeration, and better contact of residue with soil due to tillage has led to increased microbial activity, enzyme activity, and hetero trophic respiration of the microbes thus enhancing CO_2_ emissions in CTR1 and CTR2 (Sauvadet et al., 2018; Hao et al., 2019). The higher activity of methane oxidizing bacteria in NT has led to higher methane consumption and lower methane emissions in NTR1 and NTR2. Residue application enhanced the N_2_O emissions which might be due to the supply of additional organic N and increased substrate availability for microbial growth in general and N_2_O producing microbial communities in particular (Fan et al., 2019). NTR1 recorded 26 and 39% lower N_2_O emissions as compared to CTR1 and CTR2, respectively (Six et al., 2004; Ogle et al., 2005). This might be due to improvement in soil structure with residue application this improved structure might have reduced the formation of anaerobic microsites which promote N_2_O production due to denitrification. Whereas in CTR1 and CTR2 residues were mixed into the soil and were brought into direct contact with soil microbes, this results in the formation of O_2_ microsites (Mitchell et al., 2016) and higher residue N mineralization. A global meta-analysis study reported that, in dry climates N_2_O emissions were lower only in long term studies (10 years or more) with continuous NT (Van Kessel et al., 2013). While 38% higher N_2_O emissions were observed with short-term NT operations in dry climates (Van Kessel et al., 2013).

### 4.4. Relation between bacterial community, enzyme activity, and soil nutrients

The change in abundance and structure of soil microbial communities significantly influences nutrient cycling and improves the nutrient availability and a major role in the alleviation of soil degradation (Luo et al., 2018). This was evident from the present study as improvement in SOC, available nitrogen and phosphorus was noticed in zero tillage with residue application. Further, these results are supported also by RDA and Pearson’s correlation analysis.

To avoid the redundancy, a correlation study (Pearson’s correlation) was done among the different variables. The interlink between SOC and MBC indicated that they both together improved the microbial functions, enzyme activities, and soil quality with management practices. A correlation study (Pearson’s correlation) among all the 24 variables was done and findings revealed that the main bacterial phyla activity was correlated (positively or negatively) with SOC, available nutrients, and enzymes (Table 3). The bacterial species like Actinobacteria (*r* = 0.88, *p* < 0.05), Acidobacteria (*r* = 0.88, *p* < 0.05), Gemmatimonadetes (*r* = 0.89), and Nitrospirae (*r* = 0.85, *p* < 0.05) were positively correlated with SOC (Bissett et al., 2013; Fabian et al., 2017), which was the key component of soil quality. The positive correlation of Actinobacteria with OC is because these are the major saprophytic soil bacterial phylum, which produces extracellular hydrolytic enzymes and plays a key role in the degradation of OM such as cellulose, lignin, and chitin (Eisenlord and Zak, 2010) and nutrient cycling (Duran et al., 2016). Proteobacteria were efficient decomposers of OM hence the abundance of Proteobacteria was negatively correlated with SOC. Lower SOC content in CT as compared to NT might be due to a higher abundance of Proteobacteria in CT as compared to NT and RT. These bacteria were efficient decomposers of organic matter and were related to the decomposition of organic matter and carbon and, in turn, influenced soil enzyme activity.

The abundance of Actinobacteria, Acidobacteria, Gemmatimonadetes, and Nitrospira was positively correlated with acid phosphatase, dehydrogenase, and urease activity. Available nitrogen, phosphorus, potassium, and enzyme activities were strongly positively correlated with the phylum Actinobacteria, Gemmatimonadetes, and Nitrospira. Whereas the available N, P, K, and enzyme activities were significantly negatively correlated with Chloroflexi and Firmicutes. Hence, increase in enzyme activities under NTR1 and NTR2 might explain the higher available nutrients and microbial biomass carbon in NTR1. The enzymes such as acid phosphatase, alkaline phosphatase, dehydrogenase, and urease were significantly positively correlated with SOC, available nitrogen, and potassium (Akhtar et al., 2019). This indicates that the availability of carbon sources and soil organic matter decomposition influenced the enzyme activities. Moreover, this also indicates that enzymes play a major role in the biochemical mineralization of nutrients, hence increase in enzyme activities under NTR1 and NTR2 might explain the higher available nutrients, microbial biomass carbon and are better indicators of soil quality (Acosta-Martínez et al., 2018; Chen et al., 2021). A strong correlation was observed between N_2_O emissions and β- or γ-proteobacteria (AOB). Methane absorption was observed in this study and methane flux was negatively correlated with the abundance of *Methylobacterium*.

## 5. Conclusion

Soil bacterial diversity and community composition was strongly influenced by CA and this was revealed by lower CO_2_ emissions indicating lower respiration rates and higher enzyme activities. Oligotrophic bacteria like Acidobacteria, Verrucomicrobia, and Nitrospira, were higher in NT whereas copiotrophic bacteria like Proteobacteria, Chloroflexi, Planctomycetes, Bacteroidetes, and Firmicutes were higher in CT. Furthermore, SOC was higher in CA and this can lead to significant increase in soil available nutrients, bacterial populations and higher enzyme activities. CA also recorded lower CO_2_, and N_2_O emissions as well as higher CH_4_ oxidation. Our study suggests that CA (NT + crop residues) is an eco-friendly, sustainable agriculture practice as it would help to maintain the diversity and abundance of soil bacteria, improve soil health and reduces GHG emissions under semi-arid rainfed production systems of India.

## Data availability statement

The datasets presented in this study can be found in online repositories. The names of the repository/repositories and accession number(s) can be found in the article/Supplementary material.

## Author contributions

GP, MM, IS, KVR, MR, SK, and AI designed research. GP, MM, KSR, UK, and SA performed research. GP, MM, KSR, and BR analyzed data. GP and MM wrote the first draft of the manuscript. AS, CR, VS, JP, AB, and SC contributed critically to drafts. All authors contributed to the article and approved the submitted version.

## Funding

This study was financially supported by ICAR CRP-CA (F.No.11SS/CAP/2016/220).

## Conflict of interest

The authors declare that the research was conducted in the absence of any commercial or financial relationships that could be construed as a potential conflict of interest.

## Publisher’s note

All claims expressed in this article are solely those of the authors and do not necessarily represent those of their affiliated organizations, or those of the publisher, the editors and the reviewers. Any product that may be evaluated in this article, or claim that may be made by its manufacturer, is not guaranteed or endorsed by the publisher.

## Supplementary material

Supplementary Figure S1 Field experiment layout.

Supplementary Figure S2 Rarefaction curve **(a)** ChaoI species richness **(b)** of tillage methods and crop residues based on number of operational taxonomic units (OTUs) and sequences per treatments in different tillage and residue levels Note: 1-Conventional tillage; 2-Conventional tillage +10 cm anchored residue; 3-Conventional tillage +30 cm anchored residue; 4-Reduced tillage; 5-Reduced tillage+ 10 cm anchored residue; 6- Reduced tillage 30 cm anchored residue; 7-Zero tillage; 8-Zero Tillage +10 cm anchored residue; and 9-Zero Tillage +30 cm anchored residue.

Supplementary Figure S3 Proportion OTUs at class level of proteobacteria Note: CT-Conventional tillage; CTR1-Conventional tillage +10 cm anchored residue; CTR2-Conventional tillage +30 cm anchored residue; RT-Reduced tillage; RTR1- Reduced tillage+ 10 cm anchored residue; RTR2-Reduced tillage +30 cm anchored residue; NT-Zero tillage; NTR1-Zero Tillage +10 cm anchored residue; and NTR2-Zero Tillage +30 cm anchored residue.

Supplementary Figure S4 The relative abundance of bacterial classes in response to different conservation agricultural practices Note: CT-Conventional tillage; CTR1-Conventional tillage +10 cm anchored residue; CTR2-Conventional tillage +30 cm anchored residue; RT-Reduced tillage; RTR1- Reduced tillage+ 10 cm anchored residue; RTR2-Reduced tillage +30 cm anchored residue; NT-Zero tillage; NTR1-Zero Tillage +10 cm anchored residue; and NTR2-Zero Tillage +30 cm anchored residue.

Supplementary Figure S5 Heat map of Top 30 genera Note: 1-Conventional tillage; 2-Conventional tillage +10 cm anchored residue; 3- Conventional tillage +30 cm anchored residue; 4-Reduced tillage; 5- Reduced tillage+ 10 cm anchored residue; 6-Reduced tillage +30 cm anchored residue; 7-Zero tillage; 8-Zero Tillage +10 cm anchored residue; and 9-Zero Tillage +30 cm anchored residue.

Supplementary Figure S6 Relative proportion of plant growth promoting bacteria Note: CT-Conventional tillage; CTR1-Conventional tillage +10 cm anchored residue; CTR2-Conventional tillage +30 cm anchored residue; RT-Reduced tillage; RTR1- Reduced tillage+ 10 cm anchored residue; RTR2-Reduced tillage +30 cm anchored residue; NT-Zero tillage; NTR1-Zero Tillage +10 cm anchored residue; and NTR2-Zero Tillage +30 cm anchored residue.

Supplementary Figure S7 Relative proportion of ammonia and nitrite oxidizing bacteria Note: CT-Conventional tillage; CTR1-Conventional tillage +10 cm anchored residue; CTR2-Conventional tillage +30 cm anchored residue; RT-Reduced tillage; RTR1- Reduced tillage+ 10 cm anchored residue; RTR2-Reduced tillage +30 cm anchored residue; NT-Zero tillage; NTR1-Zero. Tillage +10 cm anchored residue; and NTR2-Zero Tillage +30 cm anchored residue.
