# Supplementary figures and images for: Soil bacterial community structure and functioning in a long-term conservation agriculture experiment under semi-arid rainfed production system
Source: Front Microbiol. 2023 Jun 15;14:1102682. doi: 10.3389/fmicb.2023.1102682 (PMC10307972; doi:10.3389/fmicb.2023.1102682)

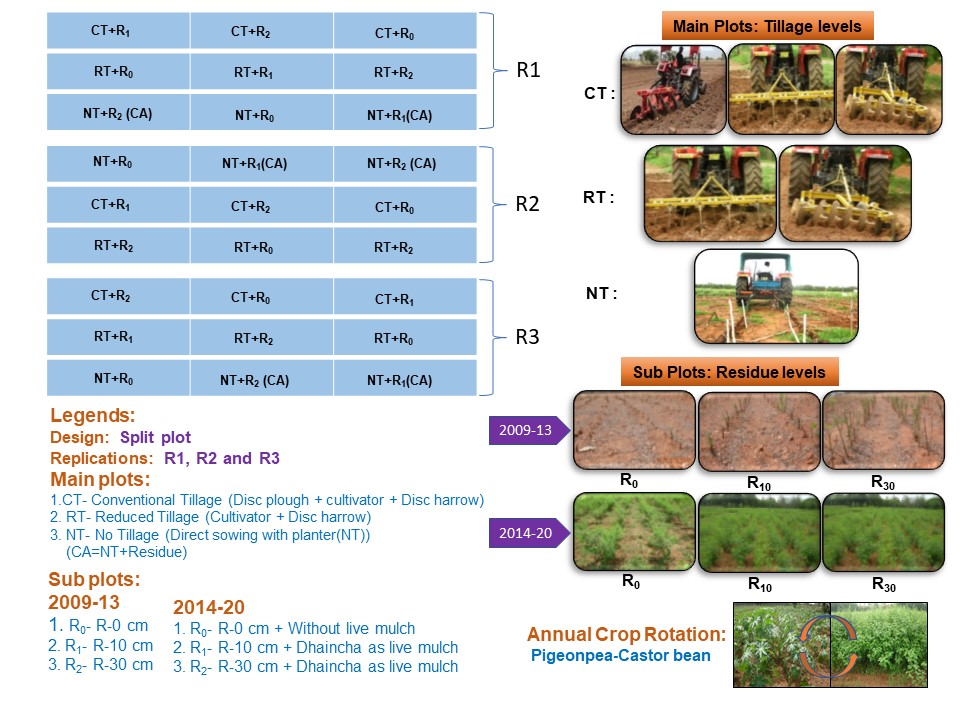

Supplement: Supplementary Figure S1 — Field experiment layout. [file Image_1.JPEG]

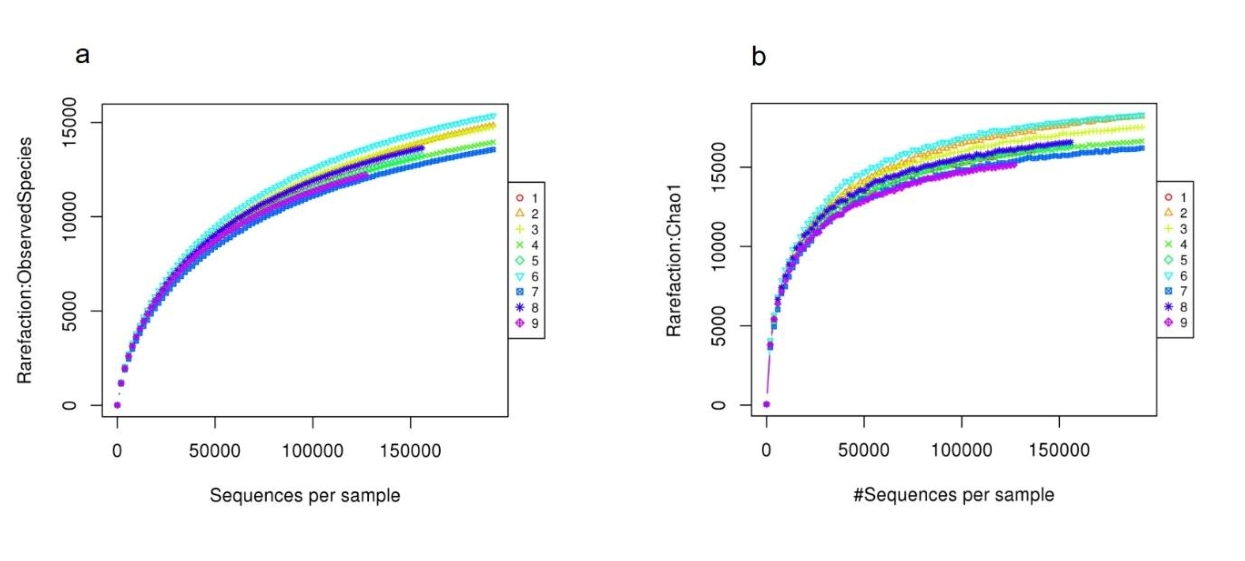

Supplement: Supplementary Figure S2 — Rarefaction curve (a) ChaoI species richness (b) of tillage methods and crop residues based on number of operational taxonomic units (OTUs) and sequences per treatments in different tillage and residue levels Note: 1-Conventional tillage; 2-Conventional tillage +10 cm anchored residue; 3-Conventional tillage +30 cm anchored residue; 4-Reduced tillage; 5-Reduced tillage+ 10 cm anchored residue; 6- Reduced tillage 30 cm anchored residue; 7-Zero tillage; 8-Zero Tillage +10 cm anchored residue; and 9-Zero Tillage +30 cm anchored residue. [file Image_2.JPEG]

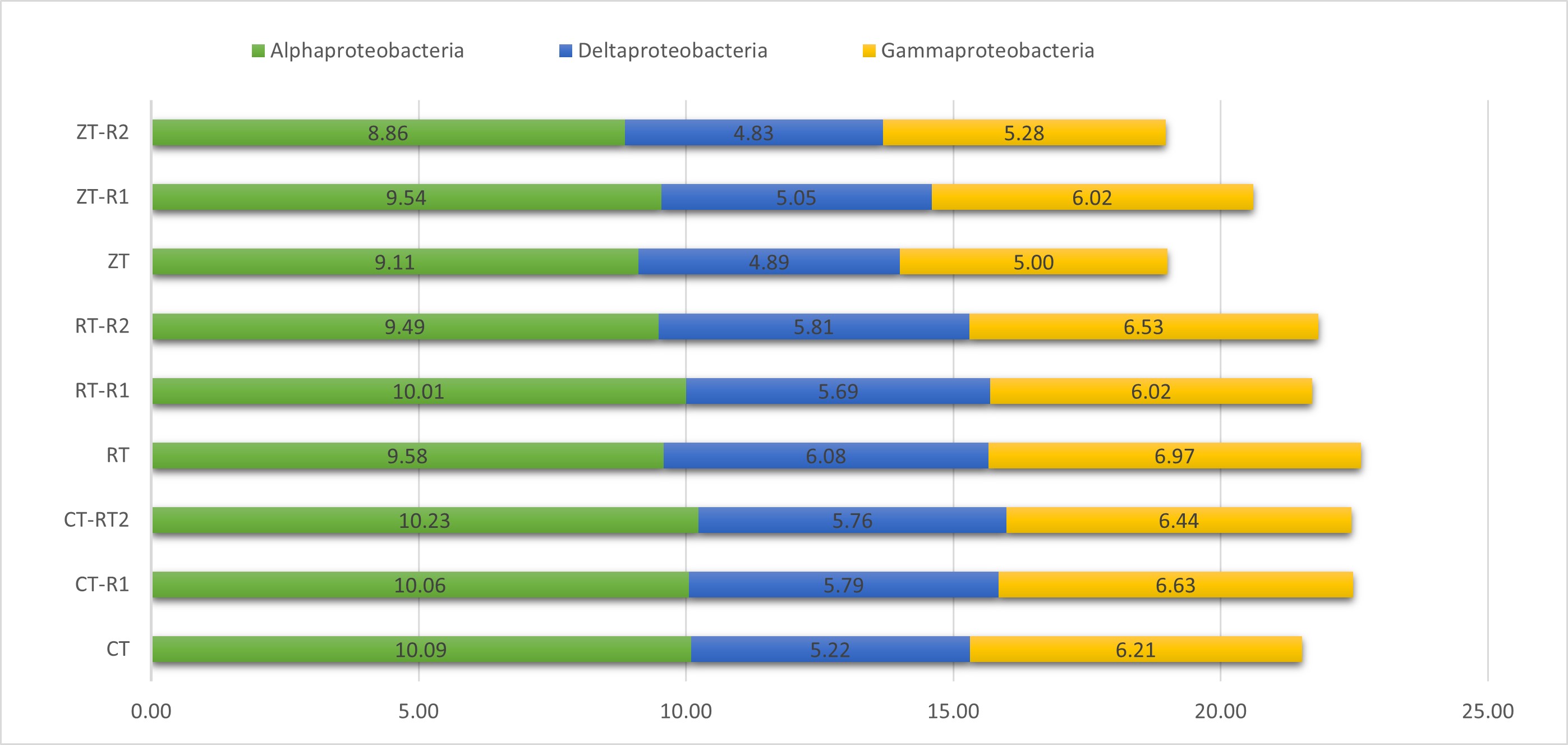

Supplement: Supplementary Figure S3 — Proportion OTUs at class level of proteobacteria Note: CT-Conventional tillage; CTR1-Conventional tillage +10 cm anchored residue; CTR2-Conventional tillage +30 cm anchored residue; RT-Reduced tillage; RTR1- Reduced tillage+ 10 cm anchored residue; RTR2-Reduced tillage +30 cm anchored residue; NT-Zero tillage; NTR1-Zero Tillage +10 cm anchored residue; and NTR2-Zero Tillage +30 cm anchored residue. [file Image_3.JPEG]

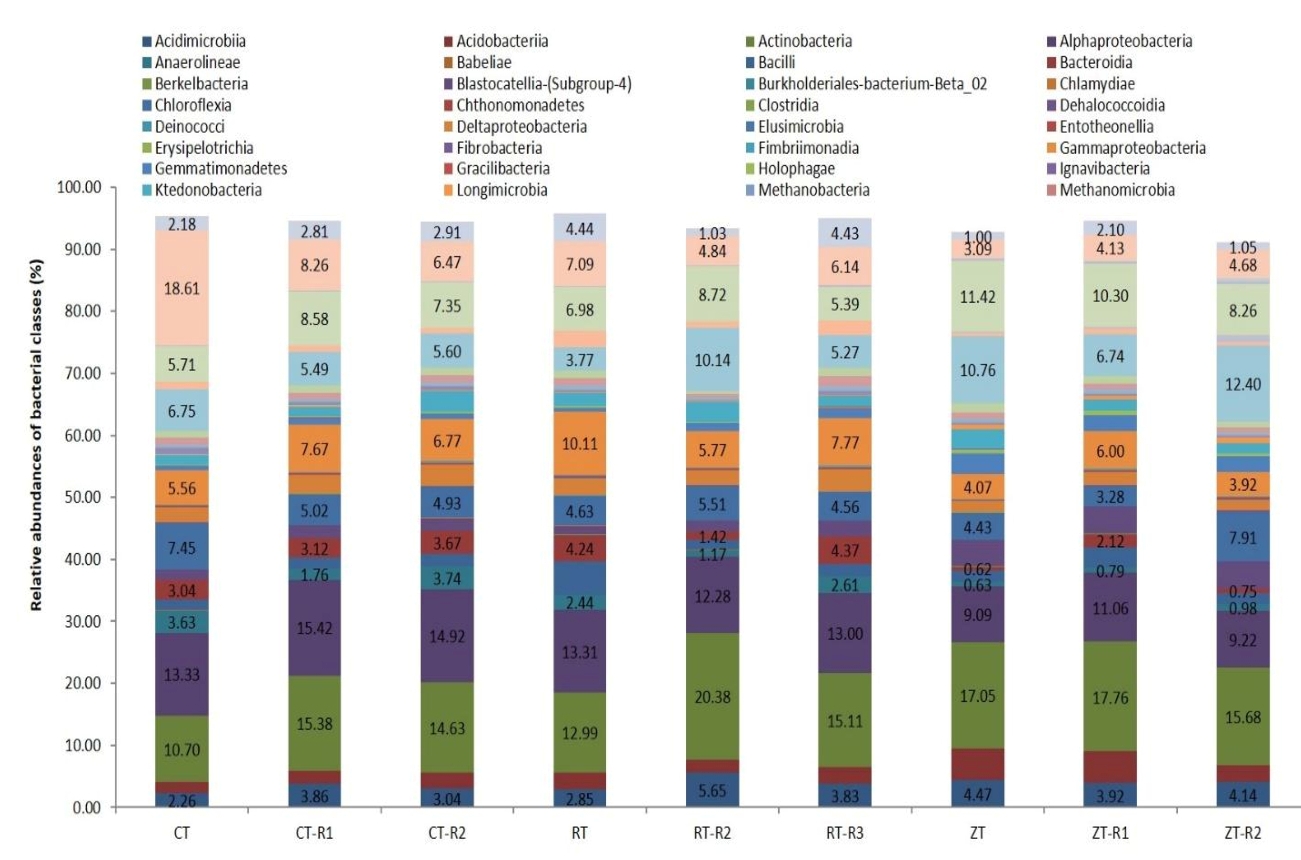

Supplement: Supplementary Figure S4 — The relative abundance of bacterial classes in response to different conservation agricultural practices Note: CT-Conventional tillage; CTR1-Conventional tillage +10 cm anchored residue; CTR2-Conventional tillage +30 cm anchored residue; RT-Reduced tillage; RTR1- Reduced tillage+ 10 cm anchored residue; RTR2-Reduced tillage +30 cm anchored residue; NT-Zero tillage; NTR1-Zero Tillage +10 cm anchored residue; and NTR2-Zero Tillage +30 cm anchored residue. [file Image_4.JPEG]

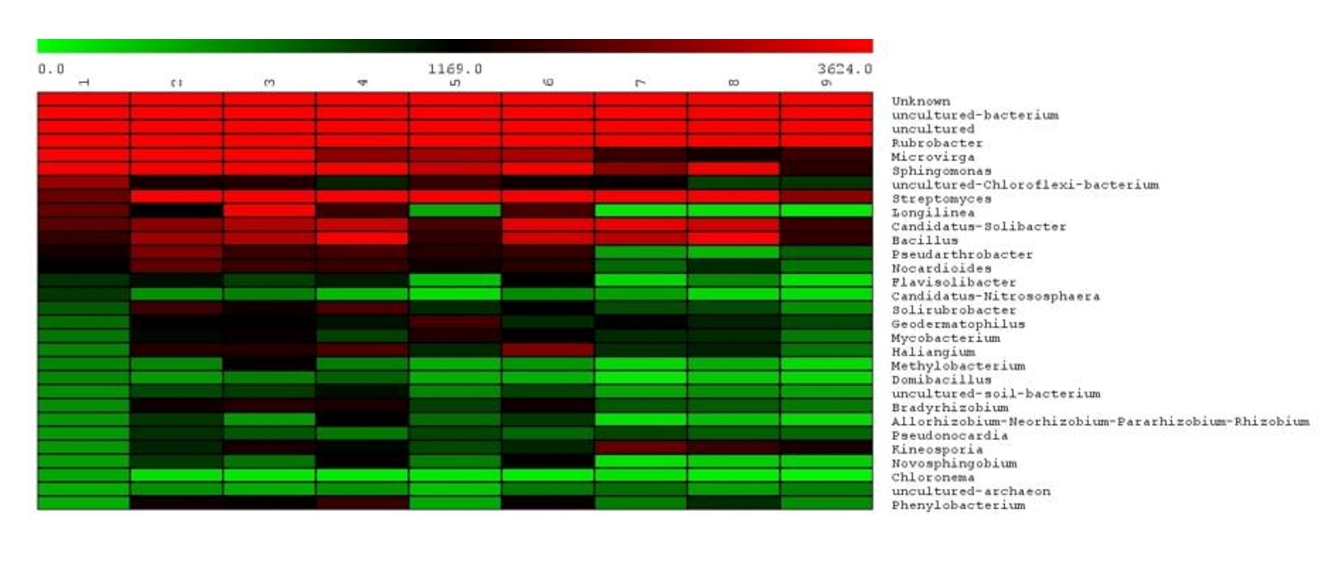

Supplement: Supplementary Figure S5 — Heat map of Top 30 genera Note: 1-Conventional tillage; 2-Conventional tillage +10 cm anchored residue; 3- Conventional tillage +30 cm anchored residue; 4-Reduced tillage; 5- Reduced tillage+ 10 cm anchored residue; 6-Reduced tillage +30 cm anchored residue; 7-Zero tillage; 8-Zero Tillage +10 cm anchored residue; and 9-Zero Tillage +30 cm anchored residue. [file Image_5.JPEG]

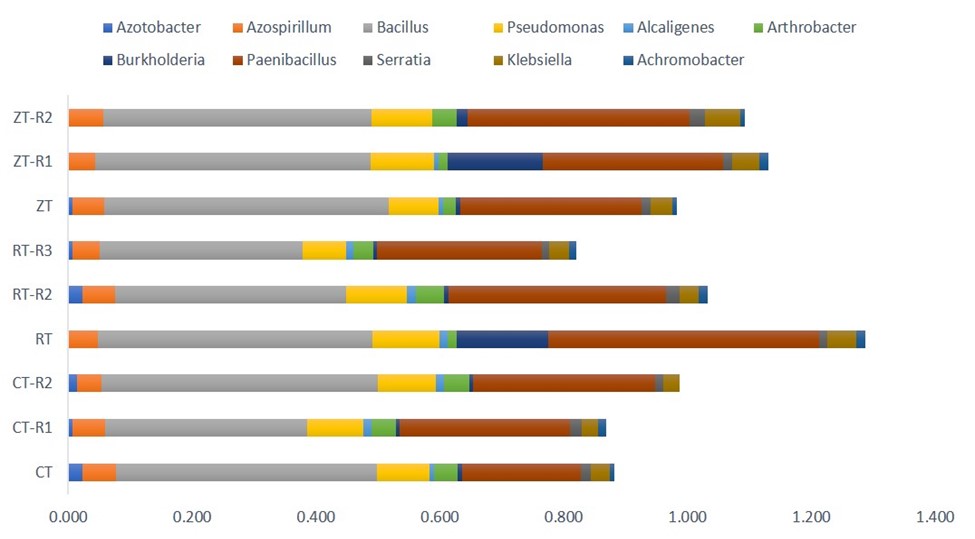

Supplement: Supplementary Figure S6 — Relative proportion of plant growth promoting bacteria Note: CT-Conventional tillage; CTR1-Conventional tillage +10 cm anchored residue; CTR2-Conventional tillage +30 cm anchored residue; RT-Reduced tillage; RTR1- Reduced tillage+ 10 cm anchored residue; RTR2-Reduced tillage +30 cm anchored residue; NT-Zero tillage; NTR1-Zero Tillage +10 cm anchored residue; and NTR2-Zero Tillage +30 cm anchored residue. [file Image_6.JPEG]

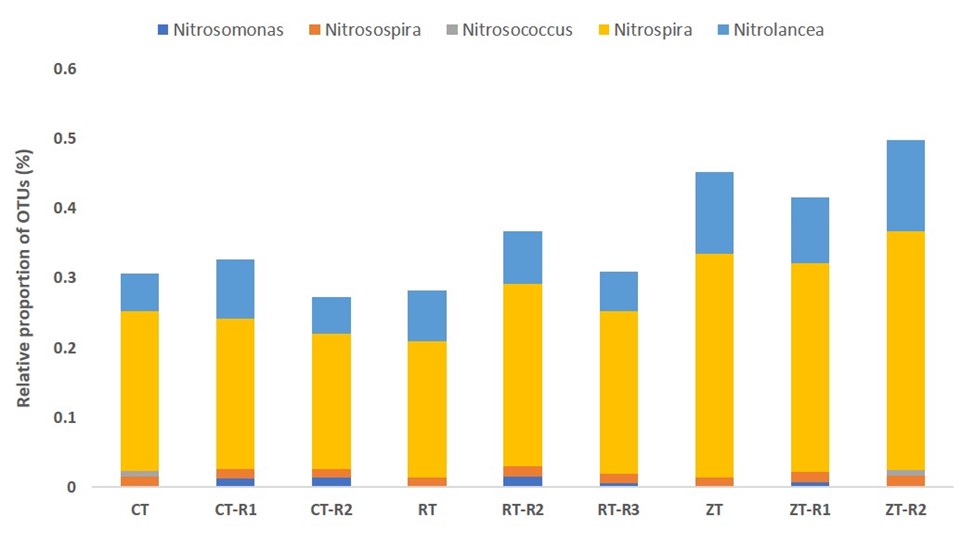

Supplement: Supplementary Figure S7 — Relative proportion of ammonia and nitrite oxidizing bacteria Note: CT-Conventional tillage; CTR1-Conventional tillage +10 cm anchored residue; CTR2-Conventional tillage +30 cm anchored residue; RT-Reduced tillage; RTR1- Reduced tillage+ 10 cm anchored residue; RTR2-Reduced tillage +30 cm anchored residue; NT-Zero tillage; NTR1-Zero. Tillage +10 cm anchored residue; and NTR2-Zero Tillage +30 cm anchored residue. [file Image_7.JPEG]
