# Supplementary material for: Soil bacterial community structure and functioning in a long-term conservation agriculture experiment under semi-arid rainfed production system
Source: Front Microbiol. 2023 Jun 15;14:1102682. doi: 10.3389/fmicb.2023.1102682 (PMC10307972; doi:10.3389/fmicb.2023.1102682)
Supplement: Supplementary file 8 [file Data_Sheet_1.pdf]

**Legends to supplementary figures**

**Fig.S1:** Field experiment layout

**Fig.S2:** Rarefaction curve (a) ChaoI species richness (b) of tillage methods and crop residues based on number of operational taxonomic units (OTUs) and sequences per treatments in different tillage and residue levels

Note: 1-Conventional tillage; 2-Conventional tillage +10 cm anchored residue; 3- Conventional tillage +30 cm anchored residue; 4-Reduced tillage; 5- Reduced tillage+ 10 cm anchored residue; 6-Reduced tillage 30 cm anchored residue; 7-Zero tillage; 8-Zero Tillage +10 cm anchored residue; and 9-Zero Tillage +30 cm anchored residue

**Fig.S3:** Proportion OTUs at class level of proteobacteria

Note: CT-Conventional tillage; CTR1-Conventional tillage +10 cm anchored residue; CTR2-Conventional tillage +30 cm anchored residue; RT-Reduced tillage; RTR1- Reduced tillage+ 10 cm anchored residue; RTR2-Reduced tillage +30 cm anchored residue; NT-Zero tillage; NTR1-Zero Tillage +10 cm anchored residue; and NTR2-Zero Tillage +30 cm anchored residue

**Fig. S4:** The relative abundance of bacterial classes in response to different conservation agricultural practices

Note: CT-Conventional tillage; CTR1-Conventional tillage +10 cm anchored residue; CTR2-Conventional tillage +30 cm anchored residue; RT-Reduced tillage; RTR1- Reduced tillage+ 10 cm anchored residue; RTR2-Reduced tillage +30 cm anchored residue; NT-Zero tillage; NTR1-Zero Tillage +10 cm anchored residue; and NTR2-Zero Tillage +30 cm anchored residue

**Fig. S5:** Heat map of Top 30 genera

Note: 1-Conventional tillage; 2-Conventional tillage +10 cm anchored residue; 3- Conventional tillage +30 cm anchored residue; 4-Reduced tillage; 5- Reduced tillage+ 10 cm anchored residue; 6-Reduced tillage +30 cm anchored residue; 7-Zero tillage; 8-Zero Tillage +10 cm anchored residue; and 9-Zero Tillage +30 cm anchored residue

**Fig. S6:** Relative proportion of plant growth promoting bacteria

Note: CT-Conventional tillage; CTR1-Conventional tillage +10 cm anchored residue; CTR2-Conventional tillage +30 cm anchored residue; RT-Reduced tillage; RTR1- Reduced tillage+ 10 cm anchored residue; RTR2-Reduced tillage +30 cm anchored residue; NT-Zero tillage; NTR1-Zero Tillage +10 cm anchored residue; and NTR2-Zero Tillage +30 cm anchored residue

**Fig. S7:** Relative proportion of ammonia and nitrite oxidizing bacteria

Note: CT-Conventional tillage; CTR1-Conventional tillage +10 cm anchored residue; CTR2-Conventional tillage +30 cm anchored residue; RT-Reduced tillage; RTR1- Reduced tillage+ 10 cm

anchored residue; RTR2-Reduced tillage +30 cm anchored residue; NT-Zero tillage; NTR1-Zero Tillage +10 cm anchored residue; and NTR2-Zero Tillage +30 cm anchored residue
