# Supplementary material for: Soil bacterial community structure and functioning in a long-term conservation agriculture experiment under semi-arid rainfed production system
Source: Front Microbiol. 2023 Jun 15;14:1102682. doi: 10.3389/fmicb.2023.1102682 (PMC10307972; doi:10.3389/fmicb.2023.1102682)
Supplement: Supplementary file 9 [file Table_1.docx]

**Table S1:** Agronomic practices followed different tillage treatments in pigeonpea–castor cropping systems

|  | | | | | |
| --- | --- | --- | --- | --- | --- |
| Crop | Month | Operation | Conventional tillage | Reduced tillage | Zero tillage |
| Pigeonpea | April/May June, June (second fortnight depending on rainfall) July | Land Preparation  Sowing + basal dose of fertilizer + pre- emergence herbicide Pre-emergence herbicide Inter-cultivation | Disk plowing once Cultivator once Disk harrow once  Sowing, 25:50:50 kg NPK/ha | -  Cultivator once Disk harrow once  Sowing, 25:50:50 kg NPK/ha, Pendimethalin | -  Sowing, 25:50:50 kg NPK/ha, Pedimethalin |
|  | July | Intercultivation | Bullock pair+Hand weeding | - | - |
|  | August | Intercultivation/Post emergence herbicides | Bullock pair+ Hand weeding | Quizalofop–p-ethyl | Quizalofop–p-ethyl |
|  | September | Intercultivation | Bullock pair + Hand weeding | Bullock pair +Hand weeding |  |
|  | October and November | Plant Protection | Need based | Need based | Need based |
|  | January | Harvesting | Harvesting | Harvesting | Harvesting |
| Castor | April/May June (second fortnight depending on rainfall) July | Land Preparation  Sowing + basal dose of fertilizer + pre emergence herbicide Pre emergence herbicide inter-cultivation | Disk plowing once Cultivator once Disk harrow once  Sowing, 25:50:50 kg NPK/ha | -  Cultivator once Disk harrow once  Sowing, 25:50:50 kg NPK/ha, Pendimethalin | -  Sowing, 25:50:50 kg NPK/ha, Pedimethalin |
|  | July | Intercultivation | Bullock pair+Hand weeding | - | - |
|  | August | Intercultivation/Post emergence herbicides | Bullock pair+Hand weeding | Quizalofop–p-ethyl | Quizalofop–p-ethyl |
|  | September | Inter cultivation  Top dressing | Bullock pair+Hand weeding  N fertilizer 25 kg ha−1 | Bullock pair+ Hand weeding  N fertilizer 25 kg ha−1 | N fertilizer 25 kg ha−1 |
|  | October and November | Plant Protection | Need based | Need based | Need based |
|  | January | Harvesting as per treatments | Harvesting as per treatments | Harvesting as per treatments | Harvesting as per treatments |
