# Supplementary material for: Soil bacterial community structure and functioning in a long-term conservation agriculture experiment under semi-arid rainfed production system
Source: Front Microbiol. 2023 Jun 15;14:1102682. doi: 10.3389/fmicb.2023.1102682 (PMC10307972; doi:10.3389/fmicb.2023.1102682)
Supplement: Supplementary file 10 [file Table_2.docx]

**Table S2:** Summary of Illumina-HiSeq®data

| **Treatments** |  | **No. of reads** | **Data in mega base pairs (MBs)** | **GC (%)** | **Total consensus sequences** | **Phred Score** |
| --- | --- | --- | --- | --- | --- | --- |
| CT | 0 | 459331 | 229.67 | 56.99 | 324053 | 34.94 |
|  | R1 | 510532 | 255.27 | 57.22 | 373517 | 34.79 |
|  | R2 | 483320 | 241.66 | 57.06 | 351968 | 34.88 |
| RT | 0 | 534208 | 267.10 | 56.54 | 385919 | 35.03 |
|  | R1 | 396584 | 198.29 | 58.30 | 290618 | 34.36 |
|  | R2 | 569539 | 284.77 | 57.32 | 395408 | 34.39 |
| NT | 0 | 550858 | 275.43 | 58.03 | 355060 | 34.07 |
|  | R1 | 392893 | 196.45 | 57.85 | 278923 | 34.43 |
|  | R2 | 357125 | 178.56 | 58.86 | 245786 | 34.10 |
