# Supplementary material for: Soil bacterial community structure and functioning in a long-term conservation agriculture experiment under semi-arid rainfed production system
Source: Front Microbiol. 2023 Jun 15;14:1102682. doi: 10.3389/fmicb.2023.1102682 (PMC10307972; doi:10.3389/fmicb.2023.1102682)
Supplement: Supplementary file 11 [file Table_3.docx]

**Table S3:** Principal component analysis of bacteria, soil nutrients, enzymes and GHG emissions

| **Principal Components** | **PC1** | **PC2** | **PC3** | **PC4** |
| --- | --- | --- | --- | --- |
| Eigen Value | 14.244 | 7.136 | 3.8056 | 2.455 |
| Variance (%) | 52.610 | 21.619 | 10.110 | 7.258 |
| Cumulative value (%) | 52.821 | 74.441 | 84.551 | 91.810 |
| **Factor loading/eigen vector** | | | | |
| Actinobacteria | **.846** | .191 | -.049 | -.160 |
| Proteobacteria | -.382 | **.794** | .387 | .024 |
| Chloroflexi | **-.919** | .138 | -.273 | -.043 |
| Planctomycetes | .459 | .751 | -.203 | -.303 |
| Acidobacteria | **.847** | .138 | .269 | .367 |
| Bacteroidetes | -.428 | .745 | .466 | -.085 |
| Verrucomicrobia | -.054 | **.835** | -.188 | -.413 |
| Gemmatimonadetes | **.921** | -.180 | -.136 | .186 |
| Firmicutes | **-.792** | -.357 | .448 | -.140 |
| Patescibacteria | -.123 | .556 | **.781** | .113 |
| Nitrospirae | **.878** | -.312 | -.148 | .197 |
| AcidPhos | **.937** | -.117 | -.118 | -.022 |
| Alk | **.784** | -.415 | .237 | -.261 |
| Av.P | **.923** | -.212 | .143 | .110 |
| Av.N | **.943** | .108 | -.038 | -.075 |
| Av.K | **.930** | .170 | .285 | -.024 |
| Dehydrogenase | **.871** | -.273 | .257 | .113 |
| Urease | **.921** | .185 | .186 | .135 |
| OC | **.889** | .286 | -.004 | .050 |
| CO_2_ | .220 | .505 | **-.782** | -.024 |
| N_2_O | .052 | **.755** | -.291 | .536 |
| CH_4_ | -.371 | .377 | -.069 | **.820** |
| MBC | **.809** | .206 | -.016 | -.306 |
| MBN | .509 | **.792** | .089 | -.174 |

Av. N, P, K: available soil nitrogen, phosphorus and potassium respectively; SOC; Soil organic carbon, CO_2_: Carbon dioxide, N_2_O: Nitrous oxide, CH_4_: Methane, MBC: soil Microbial biomass carbon, MBN: soil Microbial nitrogen, Bold letter denotes variables with higher factor loading.
